# *Toxoplasma gondii* mitochondrial association factor 1b interactome reveals novel binding partners including Ral GTPase accelerating protein α1

**DOI:** 10.1016/j.jbc.2023.105582

**Published:** 2023-12-21

**Authors:** Cameron J. Powell, Meredith L. Jenkins, Tara B. Hill, Matthew L. Blank, Leah F. Cabo, Lexie R. Thompson, John E. Burke, Jon P. Boyle, Martin J. Boulanger

**Affiliations:** 1 Department of Biochemistry and Microbiology, University of Victoria, Victoria, British Columbia, Canada; 2 Department of Biological Sciences, University of Pittsburgh, Pittsburgh, Pennsylvania, USA; 3 Department of Biochemistry and Molecular Biology, The University of British Columbia, Vancouver, British Columbia, Canada

**Keywords:** *Toxoplasma gondii*, host-pathogen interaction, host mitochondrial association, hydrogen exchange mass spectrometry, hydrogen-deuterium exchange, isothermal titration calorimetry (ITC), molecular modelling, RalGAPα1, GTPase activating protein (GAP)

## Abstract

The intracellular parasite, *Toxoplasma gondii*, has developed sophisticated molecular strategies to subvert host processes and promote growth and survival. During infection, *T. gondii* replicates in a parasitophorous vacuole (PV) and modulates host functions through a network of secreted proteins. Of these, Mitochondrial Association Factor 1b (MAF1b) recruits host mitochondria to the PV, a process that confers an *in vivo* growth advantage, though the precise mechanisms remain enigmatic. To address this knowledge gap, we mapped the MAF1b interactome in human fibroblasts using a commercial Yeast-2-hybrid (Y2H) screen, which revealed several previously unidentified binding partners including the GAP domain of Ral GTPase Accelerating Protein α1 (RalGAPα1(GAP)). Recombinantly produced MAF1b and RalGAPα1(GAP) formed as a stable binary complex as shown by size exclusion chromatography with a *K*_d_ of 334 nM as measured by isothermal titration calorimetry (ITC). Notably, no binding was detected between RalGAPα1(GAP) and the structurally conserved MAF1b homolog, MAF1a, which does not recruit host mitochondria. Next, we used hydrogen deuterium exchange mass spectrometry (HDX-MS) to map the RalGAPα1(GAP)-MAF1b interface, which led to identification of the “GAP-binding loop” on MAF1b that was confirmed by mutagenesis and ITC to be necessary for complex formation. A high-confidence Alphafold model predicts the GAP-binding loop to lie at the RalGAPα1(GAP)-MAF1b interface further supporting the HDX-MS data. Mechanistic implications of a RalGAPα1(GAP)-MAF1b complex are discussed in the context of *T. gondii* infection and indicates that MAF1b may have evolved multiple independent functions to increase *T. gondii* fitness.

Intracellular pathogens employ diverse molecular strategies to modulate host cell signaling (1, 2), metabolism (3, 4), and cellular immunity (5, 6) to increase fitness and promote growth and survival. In some cases, this may include changes to the membrane structure and composition or spatial organization of host organelles, such as mitochondria, which are frequently observed in close association with pathogen-containing vacuoles (7, 8, 9, 10, 11, 12).

The widespread human parasite *Toxoplasma gondii* recruits host mitochondria to the parasitophorous vacuolar membrane (PVM) in a process known as host mitochondrial association (HMA) (11, 12, 13). The observation that HMA only occurs in two of the three canonical strains of *T. gondii* (types I and III) facilitated analyses of a genetic cross between HMA+ and HMA-strains that led to the identification of Mitochondrial Association Factor 1b (MAF1b) as the parasite protein responsible for HMA (13, 14). Notably, the MAF1b homolog, MAF1a, with which it shares significant sequence identity (60%) and structural homology (root mean squared deviation of 0.6 Å over 240 Cαs), does not promote HMA, and its function is currently undetermined (13, 15). MAF1a and MAF1b can be found adjacent to one another in the genome, and this tandem locus has been duplicated multiple times in the *T. gondii* lineage, with high variance in copy number and primary sequence across *T. gondii* strain types (13).

Expression of MAF1b confers a competitive growth advantage to *T. gondii* parasites in an acute *in vivo* mouse model of infection (13, 16), consistent with its designation as an important virulence factor. Moreover, mice infected with MAF1b-expressing parasites have significantly higher cyst burden than the parental type II strain, which naturally lacks MAF1b expression, and this is also associated with a distinct cytokine profile marked by proinflammatory cytokines (14, 16). Originally, it was thought that recruitment of host mitochondria by MAF1b facilitated parasite scavenging of key metabolites, such as lipids, from the host cell (11, 17). Subsequent work, however, revealed that the close association of mitochondria with the PVM actually blocks the uptake of certain host fatty acids by *T. gondii* parasites, restricting intracellular growth (18). Intriguingly, however, MAF1b expression does not appear to increase the growth or multiplication of intracellular parasites in *in vitro* models of infection (13), raising questions about the precise mechanisms by which MAF1b enhances virulence *in vivo*.

Insight into this intriguing biology has been facilitated by the identification of host protein binding partners of MAF1b that are specifically required for HMA including the host outer mitochondrial membrane (OMM) import receptor, TOM70, and the mitochondria-specific chaperone, HSPA9 that were identified through mass spectrometry analysis of MAF1b co-immunoprecipitation from host lysates (19). More recently, the MAF1 locus was shown to be required for the induction of a mitochondrial stress response characterized by the shedding of the mitochondrial outer membrane. This effect was found to also be dependent upon the OMM import receptor TOM70 (12). Moreover, it was also shown that MAF1b itself could directly interact with recombinant yeast TOM70, suggesting the possibility of direct interactions between MAF1b and TOM70 from a *T. gondii* host species. Regardless, this study largely confirms prior work implicating the mitochondrial import machinery (and specifically TOM70) as a major target of MAF1b, though how these effects translate into the nuanced *in vivo* virulence effects of this protein is unknown.

In addition to its obvious effects on host mitochondrial assortment, a consistent theme across all MAF1b studies to date is the impact it has on the host immune response during infection. This effect is also consistent with the altered cytokine levels induced by MAF1b expression *in vitro* (14) and *in vivo* (16). The correlation between HMA and altered host immune signaling has prompted speculation about a putative link between these two phenomena, based on the extensive roles mitochondria play in controlling innate and adaptive immune signaling pathways (20). Despite their shared dependence on MAF1b expression, however, it is not yet clear if the observed effects of MAF1b on host immune signaling, *in vivo* parasite growth, cyst formation and/or persistence, and HMA are all directly related to each other, or result from multiple independent MAF1b effector functions.

In this study, we performed a yeast-2-hybrid (Y2H) screen to map the *T. gondii* MAF1b interactome in human fibroblasts with the goal of identifying novel host partners that may link HMA and immunomodulation or reveal alternate functions for MAF1b. Using a variety of complementary biophysical techniques, we showed that MAF1b, but not MAF1a, forms a stable binary complex with the GAP domain of human Ral-GTPase accelerating protein α1 (RalGAPα1(GAP)). Mapping of the interface by hydrogen-deuterium exchange mass spectrometry complemented with Alphafold modeling of the complex revealed a potential role for MAF1b in disrupting RalGAPα1 signaling, which plays a role in lipid homeostasis and reorganization of the cytoskeleton.

## Results

### Yeast two hybrid reveals a previously uncharacterized network of host binding partners for MAF1b

**Figure 1:**
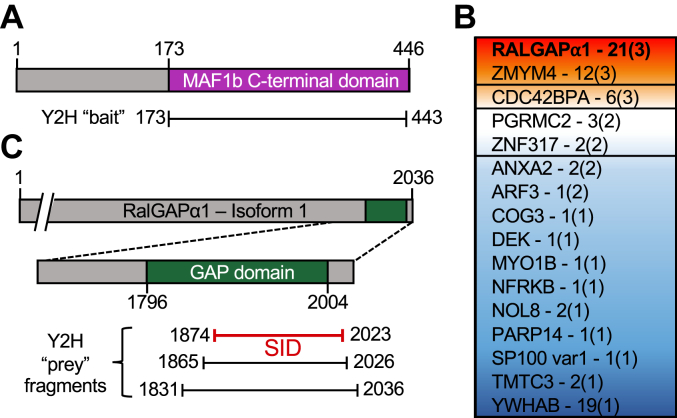
**Yeast-two-hybrid screen identifies binding partners for *T. gondii* MAF1b**. *A*, schematic of MAF1b highlighting the fragment (MAF1b (173–443), *purple*) fused to LexA for yeast two hybrid (Y2H) assay. *B*, schematic of RalGAPɑ1 highlighting the selective interaction domain (SID), which indicates the consensus interacting region of RalGAPɑ1 determined by the overlap of the clones. 21 positive clones, comprising three three unique RalGAPɑ1 fragments, where identified in the Y2H assay. Putative GAP domain and RalGAP expression construct used is coloured in *green*. *C*, Heat map of the top 16 potential MAF1b interaction partners identified *via* Y2H assay. *Colours and boxes* indicate predicted biological scores (PBS) from “A” (*high* PBS, *red*) to “D” (*low* PBS, *blue*). See Table S1 and Methods for a full list of all interactions identified, and a summary of the PBS interaction scoring system, respectively.

Previous studies have successfully relied on immunoprecipitation (IP) coupled with mass spectrometry to identify host-binding partners for MAF1b (12, 19, 21). Here we used the orthogonal approach of a yeast-two-hybrid assay (Y2H) (https://www.hybrigenics-services.com/) that is often better suited to detect transient or infrequent interactions. Briefly, the C-terminal structured region of MAF1b (residues 173–443) was fused to the C-terminus of LexA (LexA-MAF1b) (Fig. 1*A*), which was then used as bait to screen a human fibroblast cDNA library that was generated by random priming. From the Y2H screen we identified a total of 139 positive clones, comprising 76 unique prey fragments belonging to 25 putative interaction partners (Fig. 1*B* and Table S1). Of these 25 potential partners, only one has been identified in another published MAF1b Co-IP assay, desmoplakin. One single clone with a desmoplakin fragment was identified here *via* Y2H, an interaction that was assigned a low predicted biological score (PBS) (22) and labelled as a likely false positive. Boothroyd *et al* also identified an interaction between MAF1b and desmoplakin, where it was also one of the lowest ranked putative interactions (21). Interestingly, several of the top Y2H hits are known to be involved in host processes that are manipulated by the *T. gondii* parasite including cytoskeletal reorganization (ZMYM4, CDC42BPA, COG3, MYO1B) (23, 24, 25, 26), lipid homeostasis (PGRMC2, ANXA2) (27, 28), and cell morphogenesis (ZMYM4, CDC42BPA, MYO1B) (23, 24, 29). The most significant hit, however, was human Ral GTPase Accelerating Protein α1 (RalGAPα1), for which 21 positive clones were identified comprising three three unique fragments that all overlapped closely with the putative GAP domain of RalGAPα1 (hereafter referred to as RalGAPα1(GAP)) (Fig. 1, *B* and *C* and Table S1) (30). Like all GAP proteins, RalGAPα1 regulates the activity of GTPases by catalyzing (“accelerating”) hydrolysis of their bound GTP, thus switching them to an off-state (31). The primary effectors of RalGAPα1, GTPases RalA and RalB, are involved in diverse cellular processes including: mitotic regulation (32), mitochondrial fission (33), and vesicle trafficking (30, 34, 35). Notably, there was virtually no overlap between MAF1b binding partners identified in our Y2H screen and those, such as TOM70, identified in previous IP assays (12, 19, 21).

### MAF1b forms a stable binary complex with the GAP domain of human RalGAPα1

**Figure 2:**
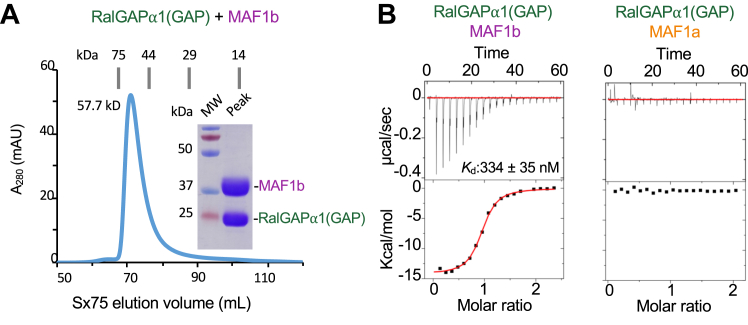
***T. gondii* MAF1b interacts with the GAP domain of human RalGAPα1.***A*, S×75 SEC profile and SDS-PAGE gel showing homogenous preparation of RalGAPα1(GAP)-MAF1b heterodimer, eluting close to the expected volume for a complex of its size. When expressed on their own, RalGAPα1(GAP) (expected: 83.7 ml *versus* Observed: 81.8 ml) and MAF1b (expected: 78.3 ml *versus* Observed: 76.0 ml) eluted from the Sx75 column as monomers. *B*, representative ITC binding isotherms following the titration of wild-type MAF1b (*left*) or MAF1a (*right*) into a cell containing RalGAPα1(GAP). Error value represents the standard deviation calculated from three independent experiments. See Table S2 for summary of thermodynamic data.

To validate the Y2H results, a twin Strep-tagged version of the putative GAP domain (residues 1807–1988) of RalGAPα1 (Fig. 1*B*) and a hexa-histidine tagged version of the structured c-terminal domain of MAF1b (residues 173–443 - hereafter simply referred to as MAF1b)), as defined from our previous structural studies (PDB: 6BXR) (15), were recombinantly produced in insect cells. Nickel purification of MAF1b followed by size exclusion chromatography (SEC) (Fig. 2*A*) revealed a single homogeneous peak with an elution profile consistent with a RalGAPα1(GAP)-MAF1b heterodimer that was further supported by SDS-PAGE analysis (Fig. 2*A* - inset). Solution binding studies using isothermal titration calorimetry (ITC) were next used to validate the Y2H and SEC results and quantitatively measure binding, stoichiometry and define the thermodynamic profile underlying complex formation. ITC was first performed on purified MAF1b and RalGAPα1(GAP), which showed a *K*_d_ of 334 ± 35 nM and a 1:1 stoichiometry, consistent with a homogeneous bimolecular complex (Fig. 2*B* – left and Table S2). Binding between MAF1b and RalGAPα1(GAP) is primarily enthalpy-driven, with a ΔH of −14.6 ± 0.3 kcal/mol, suggesting that binding is mediated by a network of favorable polar interactions. Notably, no binding was detected between the structurally conserved, but HMA-incompetent MAF1b homolog, MAF1a, and RalGAPα1(GAP) (Fig. 2*B* - right).

### Mapping the RalGAPα1(GAP)-MAF1b interface reveals the key determinants of complex formation

**Figure 3:**
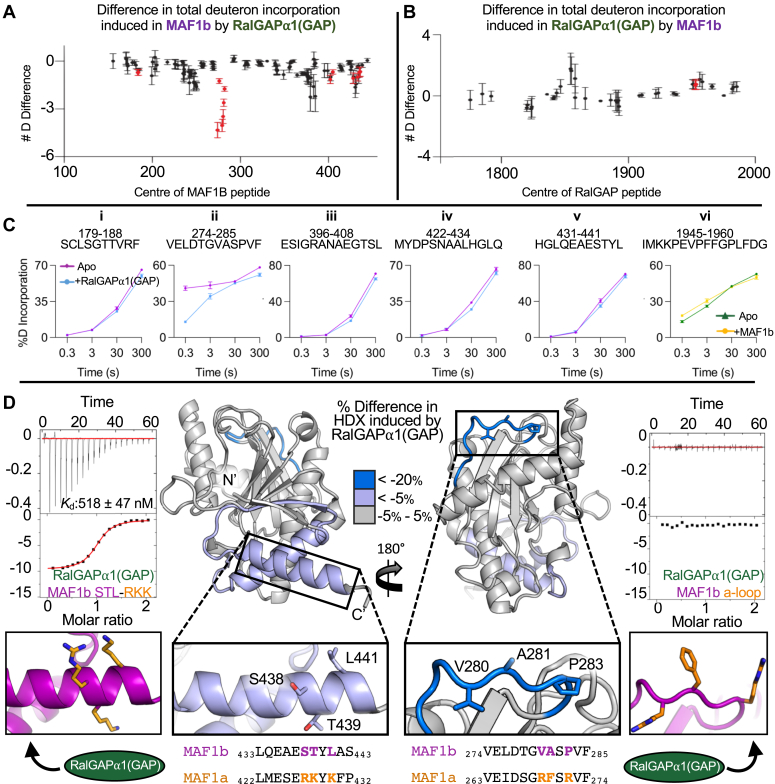
**RalGAPα1(GAP) binding mapped to a divergent surface loop on *T. gondii* MAF1b.***A*, HDX difference map showing changes in total deuteron incorporation of wild-type MAF1b, when RalGAPα1(GAP) is added. Total changes in amide exchange between conditions are summed over three time points of HDX (3s at 0 °C, 3,30 s and 300 s at 23 °C), and error bars (SD) represent independent triplicate. Peptide # corresponds to the centroid amino acid of the peptide from which a data point is obtained. For the full HDX-MS data set relating to this figure, see source data. *B*, HDX difference map showing changes in total deuteron incorporation of RalGAPα1(GAP) when wild-type MAF1b is added. Total changes in amide exchange between conditions are summed over four time points of HDX (3s at 0 °C, 3,30 s and 300 s at 23 °C), and error bars (SD) represent independent triplicate. Peptide # corresponds to the centroid amino acid of the peptide from which a data point is obtained. For the full HDX-MS data set relating to this figure, see source data. *C*, representative deuterium incorporation time courses for MAF1b and RalGAPα1(GAP) peptides showing significant differences in deuterium incorporation between apo and complex states. *D*, the difference in MAF1b HDX induced by RalGAPα1(GAP), mapped onto the crystal structure of MAF1b (PDB: 6BXR) (*middle*). Inset, left - close-up of the key C-terminal helix required for HMA and sequence alignment with corresponding helix in MAF1a, highlighting key divergent residues, with representative ITC binding isotherm following the titration of MAF1b_STL-RKK into a solution of RalGAPα1(GAP) (*left*). Inset, right-close-up of GAP-binding loop and sequence alignment of the corresponding loop in MAF1a highlighting key divergent residues, with representative ITC binding isotherm following the titration of MAF1b_a-loop into a solution of RalGAPα1(GAP) (*right*). For full HDX-MS data sets, see the attached source data excel files (Tables S3–S5).

Having validated the formation of the binary RalGAPα1(GAP)-MAF1b complex, we next mapped the interface between the two proteins using hydrogen-deuterium exchange coupled with mass spectrometry (HDX-MS). This technique quantifies the exchange of amide hydrogens with deuterium to reveal differences in secondary structure and solvent accessibility upon complex formation. HDX-MS experiments were carried out using pulses of deuterium exposure (3 s at 0 and 23 °C, and 30 and 300 s at 23 °C) with RalGAPα1(GAP) and MAF1b, individually and collectively (Fig. 3, *A* and *B* and Tables S3–S5). Significant stabilization, as quantified *via* decreased amide exchange, was observed throughout MAF1b when bound to RalGAPα1(GAP), particularly in several overlapping peptides spanning residues V274-F285. Mapping the stabilized regions of MAF1b onto our previously reported crystal structure (PDB: 6BXR) (15), revealed that residues V274-F285 are localized to a flexible solvent-exposed surface loop (hereafter designated as the “GAP binding loop”). In addition, a modest, yet still significant stabilization in the C-terminus (residues 420–440) of MAF1b (Fig. 3*A*) was also observed. Notably, the C-terminal stabilized region comprises an α-helix that was previously implicated in HMA (15). No significant decreases in deuteration were observed for RalGAPα1(GAP) upon complex formation (Fig. 3*B*), suggesting that the binding surface may either be highly dynamic or very rigid, both of which would hinder our ability to measure a significant difference in solvent exposure. However, one region (1945–1961) was significantly destabilized upon complex formation, consistent with structural rearrangement upon MAF1b binding (Fig. 3*B*).

To further dissect the interface between MAF1b and RalGAPα1(GAP), we substituted residues in the key stabilized regions of MAF1b with the analogous MAF1a residues. Double (MAF1b SL-RK) and triple (MAF1b STL-RKK) mutations in the C-term helix of MAF1b, previously shown to interfere with HMA (15), did not abrogate complex formation (Figs. 3*D* – left, S1 and Table S2), however, binding affinity was reduced from 334 nM to 518 nM. Relatively modest changes in *K*_d_ and ΔG for the mutant complexes underlie an almost complete loss of the entropic penalty associated with complex formation, accompanied by nearly commensurate reductions in ΔH (Table S2). Such a change is typically associated with the loss of non-specific hydrophobic effects during complex formation (36). These data suggest that the general reductions in HDX observed in the c-terminal region upon RalGAPα1(GAP) binding may result from allosteric conformational changes. In contrast to the C-terminal mutations, substituting just three key residues in the putative GAP binding loop of MAF1b with the homologous MAF1a residues (MAF1b_a-loop) (V280R, A281F, and P283R) completely abrogated binding with RalGAPα1(GAP) (Figs. 3*D* – right and S2) highlighting the importance of this loop region in driving complex formation.

The role(s) of specific MAF1b residues in binding to RalGAPα1(GAP) were further assessed by introducing reciprocal mutations into the relevant regions of MAF1a to test whether they were sufficient to induce assembly (Fig. S1). Mutating residues in the C-terminal helix of MAF1a to the corresponding MAF1b residues (MAF1a_RKK-STL) did not provide MAF1a with the ability to bind RalGAPα1(GAP) (Fig. S2 – left panel). Furthermore, replacing the three surface loop residues of MAF1a with the homologous MAF1b residues (MAF1a_b-loop) (R269V, F270A, and R272P) did not rescue the RalGAPα1(GAP) binding phenotype (Fig. S2 – right panel), suggesting the potential for a more complex binding mechanism involving disparate regions of the MAF1b surface that were not detected in HDX-MS experiments.

### Alphafold modelling suggests a molecular basis for RalGAPα1(GAP) selectivity for MAF1b over MAF1a

**Figure 4:**
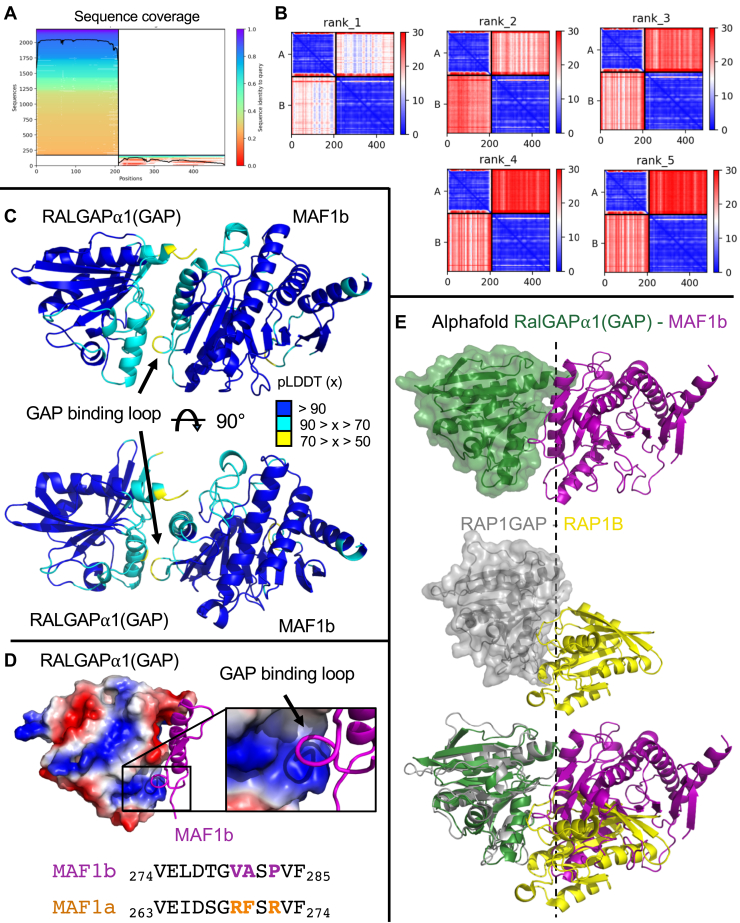
**Alphafold modeling of the RalGAP⍺1(GAP)-MAF1b complex.***A*, predicted aligned error (PAE) for Alphafold2 Multimer search of the RalGAPɑ1(GAP) domain (1807–1988) and the ordered MAF1b c-terminal domain (173–443). The colours indicate the predicted aligned error, and are coloured according to the legend. Note that the PAE plot is not an inter residue distance map or a contact map. Instead, the *red-blue colour* indicates expected distance error. The colour at (x, y) corresponds to the expected distance error in residue x’s position, when the prediction are aligned on residue y (more information can be found at https://alphafold.ebi.ac.uk/) (38, 49). *Blue* is indicative of a low PAE, and confident model prediction. *B*, multiple sequence alignment of the RalGAPɑ1(GAP) domain (1807–1988, *left*) and the ordered MAF1b c-terminal domain (173–443, *right*). *C*, Alphafold2 models from panels shown with the per-residue confidence metric predicted local distance difference test (pLDDT) coloured according to the legend. The pLDDT score varies from 0 to 100, and is an estimate of how well the prediction would agree with an experimental structure based on the local distance difference test Cα. *D*, Electrostatic surface of RalGAPɑ1(GAP) highlighting the key surface bound by the MAF1b GAP-binding loop, as modeled by Alpha Fold (*top*) and sequence alignment of corresponding loops in MAF1a and b, highlighting key divergent residues (*bottom*). *E*, comparison of the RalGAP⍺1(GAP)-MAF1b interface predicted with Alphafold2 and the previously determined co-structure of Rap1GAP-Rap1B complex (PDBID 3BRW). RalGAP⍺1(GAP) overlays with Rap1GAP with an r.m.s.d. of 1.6 Å over 158 residues. The Rap1GAP-Rap1B complex has a buried surface area of 1094 Å^2^, compared to 1277 Å^2^ for the modeled RalGAP⍺1(GAP)-MAF1b interface.

To provide additional insight into the molecular basis underpinning complex formation between MAF1b and RalGAPα1(GAP), we performed AlphaFold-multimer (https://alphafold.ebi.ac.uk/) (37, 38) modeling using ColabFold v.1.5.2 (Fig. 4) (39). Alphafold modeling is dependent on the sequence coverage of evolutionarily related sequences, which was complicated by the limited number of evolutionarily conserved homologs of MAF1b in the multiple sequence alignment (Fig. 4*A*). However, even with this limited set of multiple sequence alignments, the top ranked search model had predicted alignment error scores consistent with a stable interface (Fig. 4*B*). The highest ranked prediction of MAF1b interacting with the Ral-binding surface of RalGAPα1(GAP) had predicted alignment scores and per-residue estimate of confidence (predicted local distance difference test [pLDDT]) scores (38) consistent with good model accuracy (Fig. 4, *A*–*C*). Even though this model matches our experimental HDX-MS and mutagenesis data, there is still uncertainty in the accuracy of alphafold multimer models generated for proteins with sparse evolutionary conservation, so further structural studies will be required for high resolution insight into complex assembly.

The top model of MAF1b and RalGAPα1(GAP) in complex provided further insight into the potential binding mechanism and basis of specificity for the interaction with MAF1b over the closely related MAF1a. MAF1b and RalGAPα1(GAP) are modelled sharing an extensive interface including a BSA of 1277 Å^2^ with multiple close contacts driven by complementary charged surfaces. Notably, the binding interface on RalGAPα1(GAP) is predominantly basic (Fig. 4*D*) and would therefore repel basic residues such as those found in the loop of MAF1a that is spatially analogous to the GAP-binding loop of MAF1b. This observation provides further support for the model’s validity, as it demonstrates a correlation with results observed in our HDX-MS and ITC binding studies.

In addition to the GAP-binding loop of MAF1b, the AlphaFold model showed an α-helix comprising MAF1b residues P248-K286 in close association with RalGAPα1(GAP). No statistically significant stabilization was observed in this GAP-binding helix *via* HDX-MS, likely because it already had a stable secondary structure that could not be further stabilized by complex formation. The external surface of this helix is dominated by large, basic residues that interact strongly with the negatively charged surface of RalGAPα1(GAP) further stabilizing an interface that shows significant shape complementarity (Fig. 4*D*). Most of the residues in this helix that are oriented outwards towards RalGAPα1(GAP) are poorly conserved with those of MAF1a, including an inversion of the overall charge of this surface from negative to positive, further accounting for the specificity of the RalGAPα1(GAP)-MAF1b interaction, and revealing why mutations in the GAP-binding loop of MAF1a were not sufficient to rescue RalGAPα1(GAP) binding.

### Disruption of the RalGAPα1(GAP)-MAF1b complex does not significantly alter the HMA phenotpye

Based on the consistent profile between the selectivity of MAF1 paralogs for RalGAPα1(GAP) and the ability to induce HMA (MAF1b, unlike MAF1a, is able to induce HMA and confers an *in vivo* fitness advantage), we sought to determine the impact of disrupting MAF1-RalGAPα1 on HMA using a cell-based assay (Fig. S3, *A* and *B*). Interestingly, the MAF1b a-loop mutant, which is unable to bind RalGAPα1, was still capable of driving HMA when expressed in an HMA-null Type II strain of *T. gondii* (Fig. S3, *A* and *B*). To further probe the impact on HMA, we used an siRNA approach to knock down RalGAPα1 transcript levels followed by infection with Type II:MAF1b parasites. Knocking down RalGAPα1 proved challenging at standard siRNA concentrations. Therefore, the concentration of siRNA was increased to 40 nM, which can be toxic to host cells and increases off-target effects. Treatment with 40 nM siRNA was successful at knocking down RalGAPα1 transcript and protein levels by 45 to 60% (Fig. S4, *A*–*C*). Following 48 h of knockdown, cells were infected with Type II:MAF1b parasites. After 24 h, the percent of HMA+ vacuoles was significantly less (∗∗*p* = 0.0024 and ∗∗∗*p* = 0.0008) in the siRNA-treated cells (Fig. S4, *D* and *E*) compared to vehicle treatment. However, the number of HMA+ vacuoles also significantly decreased in the non-target siRNA (Cytophilin B) control-treated cells (Fig. S4, *A*–*E*), suggesting that the impairment of HMA may have been an artifact of high siRNA dosage. Taken together, these data indicate that, while RalGAPα1 knockdown decreases the HMA efficiency of Type II:MAF1b parasites, it is difficult to establish significance relative to off target effects related to high siRNA toxicity. Thus, formation of the RalGAPα1(GAP)-MAF1b complex may support a neofunctionalized role for MAF1b.

To further probe the significance of the MAF1b-RalGAPα1 interaction, we ectopically expressed GFP-MAF1a/b [as in (19)] (including the MAF1a/b GAP binding loop mutants described above), fused to an N-Terminal GFP tag (pcDNA3.1/NT-GFP-TOPO). Consistent with the functional studies above, the ectopically expressed MAF1b_a-loop mutant was still able to co-localize with host mitochondria 24 h post transfection in U2OS cells, and the MAF1a b-loop mutant did not induce MAF1a co-localization with the host mitochondria, remaining largely cytosolic (Fig. S5*A*). Again, this indicates that the GAP-binding region of MAF1b is not required to mediate interactions with host mitochondria. We next quantified the differences between GFP-MAF1b and GFP-MAF1b a-loop co-localization with RalGAPα1 in the nucleus of the cell expressing GFP-MAF1, in the mitochondria of the cell expressing GFP-MAF1, and in the mitochondria of a neighboring cell not expressing GFP-MAF1. It was hypothesized that if the MAF1b-RalGAPα1 interaction could be recapitulated in host cells, differences could be identified between MAF1b and MAF1b_a-loop co-localization with RalGAPα1. Notably, no significant difference was observed between RalGAPα1 localization in MAF1b-transfected and MAF1b a-loop mutant-transfected cells and no difference in RalGAPα1 localization when compared to a non-MAF1-expressing cell (Fig. S5, *B*–*D*) suggesting that MAF1b did not co-localize with RalGAPα1 in host cells. The latter result is at odds with the abundance of genetic (Y2H) and biophysical (ITC, HDX-MS) data. This discrepancy may reflect experimental limitations of the assay in localizing the MAF1b-RalGAPɑ1 interaction using fluorescence and a polyclonal antibody for such an abundant and widely distributed target protein. Other approaches such as immunogold labeling/electron microscopy or other high-resolution imaging methods may be required to adequately observe these interactions *in situ*.

## Discussion

*T. gondii* MAF1 paralogs are encoded by a multicopy locus that varies in gene number and content across *T. gondii* strains and in related species (13). The loci typically harbor two paralogs: MAF1a, which has an as-yet unknown function, and appears to have no impact on HMA (13, 15) and MAF1b, which is required for the long-observed phenomenon of host mitochondrial association (HMA) in *T. gondii* (13, 14). MAF1b is also an important parasite virulence factor and alters host cell immune signaling *in vitro* (12) and *in vivo* (16). While it has been shown that MAF1b expression by *T. gondii* confers a competitive growth advantage *in vivo* (13) and alters host immune signaling (14), the precise mechanisms mediating altered *in vivo* behavior are not well understood. It is not yet clear if alterations in host immune signaling are a direct result of HMA, or if these processes represent multi-effector functions of MAF1b.

### Identification and validation of a novel RalGAPα1(GAP)-MAF1b complex

Using a Y2H screen with MAF1b as “bait”, we identified several new host-derived binding partners. Notably, there was almost no overlap between MAF1b binding partners identified here, *via* Y2H, and those previously observed *via* IP experiments (12, 19, 21). Intriguingly, several of the host proteins identified in the Y2H screen play key roles in various host processes that are manipulated by the *T. gondii* parasite (23, 24, 25, 26, 27, 28, 29). Based on the number of positive clones and overlapping peptides, the highest priority Y2H hit was human RalGAPα1, and more specifically its GAP domain, *via* which RalGAPα1 regulates the activity of key signaling proteins RalA and RalB to influence a wide array of cellular processes (31).

To validate the Y2H data, we first recombinantly produced both MAF1b and RalGAPα1(GAP) and showed that the proteins formed a stable heterodimer by SEC. Biophysical characterization using ITC confirmed the 1:1 stoichiometry with a K_d_ of 334 nM. Using HDX-MS, we further validated the complex by identifying specific regions that become ordered upon complex formation including an MAF1b surface loop, which we designated as the “GAP-binding loop”. Substitution of three residues in the GAP-binding loop of MAF1b with the analogous residues from MAF1a (MAF1b_a-loop) was sufficient to abrogate binding consistent with our ITC data that showed only MAF1b, and not MAF1a, was able to form a complex with RalGAPα1(GAP). These data are also consistent with a model of the complex generated using AlphaFold-multimer, which shows that substituted residues from MAF1a are physically and electrostatically incompatible with binding the interface surface of RalGAPα1(GAP). Despite the crucial contribution of the GAP-binding loop to complex formation, complementary mutations in MAF1a (MAF1a_b-loop) were not sufficient to enable binding to RalGAPα1(GAP), likely due to an additional GAP-binding helix (MAF1b P248-K286). The positively charged GAP-binding α-helix of MAF1b comprises the majority of the interface with RalGAPα1(GAP) by forming an extensive network of charge-based interactions with a complimentary acidic pocket on the surface of the GAP domain. Notably, all the external residues (oriented outwards towards RalGAPα1(GAP)) of this helix are poorly conserved with those of MAF1a, including a significant charge inversion of the helix, from strongly basic (MAF1b) to moderately acidic (MAF1a). The mutagenesis and binding data presented herein, combined with *in silico* modelling of the corresponding atomic structure, reveal the underlying basis for RalGAPα1(GAP) selectivity in binding MAF1b over MAF1a, while highlighting key roles for the GAP-binding loop and GAP-binding helix in forming this interface.

### Mechanistic implications of a RalGAPα1(GAP)-Maf1b complex

As described above, our functional analysis failed to link residues critical for MAF1b interaction with RalGAPα1 with MAF1b-driven HMA (Figs. S3 and S4), and we were unable to demonstrate that RalGAPα1 was relocalized in a MAF1b-dependent manner (Fig. S5). While these observations may reflect technical limitations of the assay (*e.g.* the use of a single time point or a specific cell type) or the activation of compensatory pathways, our data suggests that the ability of MAF1b to bind RalGAPα1(GAP) is likely responsible for alterations to host cell biology separate from the HMA phenotype. There are challenges associated with detecting re-localization of a protein like RalGAPα1 by MAF1b since both this *T. gondii* protein and RalGAPα1 co-localize with mitochondria. Moreover, the amount of RALGAPα1 in the host cells appears to be much higher than the amount of MAF1b found in the parasitophorous vacuolar membrane, and therefore the amount of protein relocalized by MAF1b may be small in comparison and therefore difficult to detect due to signal to noise issues.

Intriguingly, analysis of the AlphaFold model of a MAF1b-RalGAPα1(GAP) heterodimer revealed a possible functional mechanism whereby MAF1b may disrupt GAP-mediated signaling from RalGAPα1(GAP). There are currently no published structures of a Ral GTPase/RalGAP complex with which to assess the functional impact of binding MAF1b, so we leveraged the most closely related complex for which there is structural data available, human RapGAP in complex with Rap GTPase (PDBID 3BRW) (40). By overlaying RalGAPα1(GAP) from our AlphaFold model with the GAP domain of human RapGAP in complex with Rap GTPase (Fig. 4*D*), we showed that the surface of RalGAPα1(GAP) bound by MAF1b shares significant overlap with the expected interface between RalGAPα1 and a Ral GTPase, including complete occlusion of the critical catalytic asparagine residue of RalGAPα1 (N-1950) (31). Thus, formation of a RalGAPα1(GAP)-MAF1b complex likely prevents binding and subsequent deactivation of a Ral GTPase by RalGAPα1, which would result in constitutively active RalA or RalB within the host cell. Ral GTPases regulate a wide array of cellular processes including autophagy and vesicle trafficking thus, their constitutive activation would likely have profound impacts on host cell signaling and metabolism.

The possibility that *T. gondii* MAF1b modulates host RalGAPα1 activity, which, in turn, regulates the activity of key host signaling proteins, RalA and RalB is an intriguing prospect due to the wide array of potential effects on the host cell. Notably, several changes in host cell function induced by *T. gondii* infection overlap with the effects of constitutive activation of RalA or RalB expected to result from MAF1b forming a complex with RalGAPα1 (31). For example, *T. gondii* actively promotes autophagy in host cells, in a Beclin1-dependent manner, in order to facilitate nutrient acquisition and parasite growth (3, 4, 18), while activated RalB is required for induction of autophagy, *via* the assembly of the Beclin1/ULK1/VPS34 autophagy initiation complex (41). Furthermore, Exo84, the direct RalB effector by which autophagy is initiated, is enriched in eluates of mouse embryonic fibroblasts following immunoprecipitation with MAF1b (21). *T. gondii* also suppresses host cell apoptosis to promote its own survival (42), and expression of MAF1b in a type II *T. gondii* background altered host expression of type-1 interferon-induced genes (14). While a definitive mechanism has not been established for either of these processes, it is interesting to note that chronic activation of RalB has also been shown to restrict activation of apoptotic programs, and drive activation of type-1 interferon (43). While the Ral GTPases are involved in diverse signaling pathways throughout the host cell, it is also important to note that MAF1b is primarily found anchored to the PV membrane, and within the parasite itself, during *T. gondii* infection. As such, it is possible that any functional consequences of the MAF1b-RalGAPα1 interaction are more subtle, and thus more difficult to detect, being that they may be restricted to the immediate vicinity of the PV rather than distributed throughout the entire cell.

The interaction between MAF1b and RalGAPα1(GAP) reported here reveals new insights into the molecular crosstalk between *T. gondii* and its hosts. As an obligate intracellular pathogen, *T. gondii* has developed sophisticated mechanisms to control a wide variety of host processes enabling invasion, replication, dissemination and increasing overall fitness. The potential for MAF1b to disrupt RalGAPα1 associated signaling process offers an intriguing host manipulation strategy that may contribute to the increased fitness observed in parasites expressing MAF1b.

## Experimental procedures

### Cloning, protein production and purification

*T. gondii* MAF1a, MAF1a_b-loop, MAF1a_RKK-STL, MAF1b, MAF1b_a-loop, and MAF1b_STL-RKK – Clones encoding amino acids T159-D435 of MAF1a (WT, R269V/F270A/R272P [b-loop], and R427S/K428T/K430L [RKK-STL]) and S173-D443 of MAF1b (WT, V280R/A281F/P283R [a-loop], S438R/L441K [SL-RK], and S438R/T439K/L441K [STL-RKK]) were codon optimized for expression in *E. coli* and synthesized by GenScript. Amino acid sequences for the two wild-type MAF1 homologues were derived from the RH strain of *T. gondii*. Genes were subcloned into an engineered vector encoding a TEV protease cleavable N-terminal hexa-histidine tag. MAF1 constructs were produced and purified as previously reported (15). Briefly, proteins were expressed in BL21 DE3 cells overnight at 30 °C and purified with Ni-affinity chromatography. Affinity tags were cleaved overnight with TEV protease, and further purified using size exclusion chromatography (SEC) with a Superdex 75 or 200 in 20 mM Hepes pH 7.5 to 8.0, 150 mM NaCl and 1 mM DTT. RalGAPα1(GAP) – Clones encoding amino acids T1807-P1988 of Human RalGAPα1 (designated as RalGAPα1(GAP)) (Accession: NP_055805.1) were codon optimized for expression in insect cells and synthesized by GenScript. Genes were subcloned into an engineered vector encoding a TEV protease cleavable N-terminal twin-strep-II tag. RalGAPα1(GAP) was expressed in *T. ni* cells with or without MAF1b_WT. After 72 h, the cells were lysed by sonication in 20 mM Hepes pH 8.0, and 150 mM NaCl, purified with streptactin affinity chromatography, and cleaved overnight with TEV protease. The RalGAP (±MAF1b) was further purified using size exclusion chromatography (SEC) with a Superdex 75 16/600 column in 20 mM HEPES pH 7.5, 150 mM NaCl and 1 mM DTT. Molecular weights of all proteins and complexes were determined by first establishing a standard curve using “low MW Gel Filtration Calibration kit” (GE cat no. 28–4038–41), and then calculating based on elution volume, using the formula: Log[molecular weight (Da)] = [Elution volume (ml) - 229.33]/−33.76.

### Yeast two-hybrid analysis

Yeast two-hybrid screening was performed by Hybrigenics Services, S.A.S., Paris, France (http://www.hybrigenics-services.com).

The coding sequence for amino acids 173 to 443 of *maf1b* was PCR-amplified and cloned into pB27 as a C-terminal fusion to LexA (LexA-MAF1b). The construct was checked by sequencing the entire insert and used as a bait to screen a human fibroblast cDNA library, that was generated by random priming, constructed into pP6. pB27 and pP6 derived from the original pBTM116 (44) and pGADGH (45) plasmids, respectively. 86 million clones (9-fold the complexity of the library) were screened using a mating approach with YHGX13 (Y187 ade2-101::loxP-kanMX-loxP, matα) and L40ΔGal4 (mata) yeast strains as previously described (46). 139 His+ colonies were selected on a medium lacking tryptophan, leucine and histidine. The prey fragments of the positive clones were amplified by PCR and sequenced at their 5′ and 3′ junctions. The resulting sequences were used to identify the corresponding interacting proteins in the GenBank database (NCBI) using a fully automated procedure. A confidence score (PBS, for Predicted Biological Score) was attributed to each interaction as previously described (22).

### Isothermal titration calorimetry

Purified *T. gondii* MAF1a, MAF1a_b-loop, MAF1a_RKK-STL, MAF1b, MAF1b_a-loop, MAF1b_SL-RK, MAF1b_STL-RKK, and RalGAPα1(GAP) were dialyzed separately against 20 mM Hepes pH 7.5, 150 mM NaCl and 1 mM TCEP at 4 °C overnight. All ITC experiments were carried out at 20 °C on a MicroCal iTC200 instrument (GE Healthcare). The sample cell contained RalGAPα1(GAP) (15 μM), and MAF1 (150 μM) was added in 19 injections of 2 μl each. Data was processed using Origin software (MicroCal) and the dissociation constants (*K*_d_) were determined using a one-site model. Figures are of a single experiment but are representative of three independent experiments.

### HDX-MS

HDX exposures were conducted on RalGAPα1(GAP) and MAF1b, individually and collectively, in 50 μl reactions with a final concentration of 2.0 μM of each protein per sample. Reactions were initiated by the addition of 45 μl of D_2_O buffer (10 mM Hepes pH 7.5, 50 mM NaCl, 0.5 mM TCEP, 97% D_2_O) to 5 μl of protein solution to give a final concentration of 87% D_2_O. Exchange was carried out for 3 s at 0 °C and for 3 s, 30 s, and 300 s at 23 °C. Exchange was terminated by the addition guanidine-HCl (final 0.6 M) and 0.8% formic acid. Experiments were carried out in triplicate. Samples were immediately frozen in liquid nitrogen and stored at −80 °C until mass analysis. Protein samples were rapidly thawed and injected onto an ultra-performance liquid chromatography (UPLC) system at 2 °C. The protein was run over two immobilized pepsin columns (Applied Biosystems; Porosyme, 2–3131–00) at 10 °C and 2 °C at 200 μl/min for 3 min and the peptides collected onto a VanGuard Precolumn trap (Waters). The trap was subsequently eluted in line with an ACQUITY 1.7 μm particle, 100 × 1 mm^2^ C18 UPLC column (Waters), using a gradient of 3 to 35% B (buffer A, 0.1% formic acid; buffer B, 100% acetonitrile) over 11 min immediately followed by a gradient of 35 to 80% B over 5 min. Mass spectrometry experiments were performed on an Impact II QTOF (Bruker) acquiring over a mass range from 150 to 2200 m/z using an electrospray ionization source operated at a temperature of 200 °C, and a spray voltage of 4.5 kV. Peptides were identified from the non-deuterated samples of MAF1b or RalGAPα1(GAP) alone using data-dependent acquisition following tandem MS/MS experiments (0.5 s precursor scan from 150-2000 m/z; twelve 0.25 s fragment scans from 150-2000 m/z). MS/MS datasets were analyzed using PEAKS7 (PEAKS), and a false discovery rate was set at 1% using a database of purified proteins and known contaminants. The search parameters were set with a precursor tolerance of 20 ppm, fragment mass error 0.02 Da, charge states from 1 to 8.

HDExaminer Software (Sierra Analytics) was used to calculate deuterium incorporation into each peptide. All peptides were manually inspected for the correct charge state and the presence of overlapping peptides. Deuteration levels were calculated using the centroid of the experimental isotope clusters. Results are presented as relative levels of deuterium incorporation, with the only correction being applied correcting for the deuterium oxide percentage of the buffer utilized in the exchange. Differences in exchange in a peptide were considered significant if they met all of the following criteria: >5% change in exchange and >0.35 Da difference in exchange, and a *p* value < 0.05 using a two tailed Student’s *t* test. To allow for visualization of differences across all peptides, we utilized number of deuteron difference (#D) plots (Fig. 3*A*). These plots show the total difference in deuterium incorporation over the entire H/D exchange time course, with each point indicating a single peptide. The data analysis statistics for all HDX-MS experiments are in the source data according to the guidelines of (47). The mass spectrometry proteomics data have been deposited to the ProteomeXchange Consortium *via* the PRIDE partner repository (48) with the dataset identifier PXD034607. For full HDX-MS data sets, see the attached source data excel files (Tables S3–S5).

### Alphafold modeling

All protein models were generated using AlphaFold2 Multimer (37) implemented in the Colabfold 1.5.2 interface available on the Google Colab platform (39) (https://colab.research.google.com/github/sokrypton/ColabFold/blob/main/AlphaFold2.ipynb). The sequence of RalGAPα1(GAP) (1807–1988) and MAF1b c-terminal domain (173–443) were used as the search input. The full set of validation statistics and multiple sequence alignment used to generate the model are shown in Figure 4, *A*–*C*. There was no consensus solution across all five models, however, the top-ranked model showed pLDDT and PAE scores consistent with a stable interface, and was experimentally supported by the HDX-MS and mutational analysis.

### Parasite cultivation and infections

TgMe49 in these experiments were regularly passed in human foreskin fibroblasts (HFFs) and incubated at 37 °C in 5% CO2. HFFs were grown in Dulbecco’s modified Eagle’s medium (DMEM) supplemented with 50 ug/ml of penicillin and streptomycin, 10% FBS, and 2 mM glutamine (cDMEM). Parasites were routinely harvested by needle passage, quantified using a hemacytometer and then used to infect host cell monolayers.

### Immunofluorescence assays and microscopy

HFFs were grown to 100% respectively, on 0.7 cm^2^ 8-well glass chamber slides (ThermoFisher Scientific) in cDMEM. Monolayers were infected at an MOI of 1 with transgenic parasites. Cells were fixed at 18 hpi with 4% paraformaldehyde for 15 min and blocked/permeabilized with blocking buffer (5% BSA, 0.1% Triton X-100, PBS). HFFs were then probed with anti-HA rat monoclonal antibody (3F10 clone, Roche) diluted to 0.1 μg/ml in blocking buffer (see above) for 1 h at room temperature while shaking. HFFs were also incubated in anti-MTCO2 mouse monoclonal antibody (Abcam; ab110258) and cells were washed with PBS. HFFs were incubated in 488 goat anti-rat and 594 goat anti-mouse secondary antibody (Life Technologies Alexa Fluor H + L) for 1 h at room temperature while shaking, followed by PBS washes. HFFs were then mounted in Vectashield mounting media (Vector laboratories) and sealed with cover glass. Slides were visualized using epifluorescence microscopy. Images were taken of the three channels: 488 (anti-HA), 594 (anti-MTCO2 and mito-RFP) and DIC/phase. Images were cropped and merged using ImageJ (NIH).

## Data availability

All data are contained within the manuscript and supporting information document.

## Supporting information

This article contains supporting information (19).

## Conflict of interest

The authors declare that they have no known competing financial interests or personal relationships that could have appeared to influence the work reported in this paper.
