# Supplementary material for: Toxoplasma gondii mitochondrial association factor 1b interactome reveals novel binding partners including Ral GTPase accelerating protein α1
Source: J Biol Chem. 2023 Dec 21;300(1):105582. doi: 10.1016/j.jbc.2023.105582 (PMC10821591; doi:10.1016/j.jbc.2023.105582)
Supplement: Supporting information [file mmc1.docx]

*Toxoplasma gondii* Mitochondrial Association Factor 1b interactome analysis reveals previously unidentified binding partners including Ral GTPase Accelerating Protein α1

**Cameron J. Powell^1^, Meredith L. Jenkins^1^, Tara B. Hill^1^, Matthew L. Blank^2^, Leah F. Cabo^2^, Lexie R. Thompson^1^, John E. Burke^1^, Jon P. Boyle^2^, and Martin J. Boulanger^1^**

**Experimental procedures**

*Cell maintenance and parasite infection* – *Tg*Me49 in these experiments were regularly passed in human foreskin fibroblasts (HFFs) and incubated at 37^o^C in 5% CO_2_ and grown in Dulbecco’s modified Eagle’s medium (DMEM) supplemented with 50 μg/ml of penicillin and streptomycin, 10% FBS, and 2mM glutamine (cDMEM).

*siRNA knockdown of RalGAPα1 and impact on HMA -* U2OS cells were treated with 40nM of RalGAPα1 (set 2) single target siRNA or RalGAPα1 siRNA smart pool (RG pool) in cDMEM as described previously (1). RNA was collected at 48 hours and qPCR analysis was performed or cells were lysed in Pierce IP lysis buffer and boiled in 1x SDS. Samples were resolved on 10% SDS-PAGE gel and transferred to nitrocellulose. Both RalGAPα1 (Sigma-Aldrich Atlas Antibodies #HPA000851) and αTubulin (Cell Signaling Technology #2144) antibodies were used for western analysis. For immunofluorescence cells were fixed, permeabilized, and visualized using fluorescence microscopy with the indicated antibodies (see below). To determine the impact of RalGAPα1 knockdown HMA+ and HMA- vacuoles were counted (n=3, 50 per treatment group) using fluorescence microscopy and data analyzed using a t-test comparing treatment to vehicle.

*Expression of MAF1 mutants and transgenic parasites –* Parental plasmid used for all *Tg*MAF1RHb1 and TgMAF1Rha1 constructs contains the *HXGPRT* gene. The GAP-binding loop was swapped between the RHb1 and RHa1 constructs using splicing by overlap extension (SOE) PCR. All constructs were confirmed by sanger sequencing and all constructs contained the endogenous *Tg*MAF1RHb1 promoter followed by the start codon, signal peptide, and a N-terminal hemagglutinin (HA) epitope tag. Transgenic lines were generated using *Tg*Me49∆HXGPRT:Luciferase parasites that were transfected with 55 μg of DNA linearized with *Hind*III. A T25 flask of *Tg*Me49∆HXGPRT:Luciferase parasites was scraped and passed through a 25 and 27 gauge needle followed by centrifugation for 10 minutes at 800xg. 2x10^7^ parasites were re-suspended in Cytomix (120 mM KCl; 0.15 mM CaCl_2_; 10 mM KPO_4_; 25 mM Hepes, 2mM EDTA, 5mM MgCl_2_; pH to 7.6), GSH and ATP. Parasites were electroporated with 1.6Kv and 25μF. Following 24 hours of growth in cDMEM, parasites were selected with mycophenolic acid (MPA) and xanthine 50 and 25 μg/mL, respectively. Selected populations were then cloned via limited serial dilution in a 96-well plate. Cloned parasites were confirmed through immunofluorescence assays (IFA) by probing for HA epitope tag.

*Immunofluorescence assays and microscopy on parasite infected cells –* HFFs were grown to 100% and 60% confluency, respectively, on 0.7 cm^2^ 8-well glass chamber slide system (ThermoFisher Scientific) in cDMEM. Monolayers were infected at an MOI of 1 with transgenic parasites. Cell were fixed at 18 hpi with 4% paraformaldehyde for 15 min. and blocked/permeabilized with blocking buffer (5% BSA, 0.1% Triton X-100, PBS). HFFs were then probed with anti-HA rat monoclonal antibody (3F10 clone, Roche) diluted to 0.1 μg/mL in blocking buffer (see above) for 1 hour at room temperature while shaking. HFFs were also incubated in anti-MTCO2 (“Mito”: ab110258, Abcam) mouse monoclonal antibody and cells were washed with PBS. HFFs were incubated in 488 goat anti-rat and 594 goat anti-mouse secondary antibody (Life Technologies Alexa Fluor H+L) for 1 hour at room temperature while shaking, followed by PBS washes. HFFs were then mounted in Vectashield mounting media (Vector laboratories) and sealed with nail polish. Slides were visualized using epifluorescence microscopy. Images were taken of the three channels: 488 (anti-HA), 594 (anti-MTCO2 and mito-RFP) and phase contrast. Images were cropped and merged using ImageJ (NIH).

*Expression of MAF1b-GFP fusions and analysis of the impact of RalGAPα1 localization* - Mammalian GFP-MAF1 plasmids were generated using pcDNA3.1/NT-GFP TOPO plasmid cloning kit. U2OS cells were transfected with each construct by lipofection (Lipofectamine 3000, ThermoFisher). Cells were fixed and permeabilized at 24 hours post transfection and stained for RalGAPα1 (Sigma-Aldrich Atlas Antibodies #HPA000851) and mitochondria (Abcam #92824). Pixel intensity was measured in both the RalGAPα1 and mitochondria channel over the mitochondria of a MAF1-expressing cell, the nucleus of a MAF1-expressing cell, and the mitochondria of a neighboring non-MAF1-expressing cell. The ratio of RalGAPα1/mitochondria pixel intensity was measured for each of these three locations. This same procedure was performed for the MAF1b mutants. Ratios were analyzed for statistical significance using One Way ANOVA followed by Tukey’s HSD post-hoc test.

**Tables**

**Table S1**. Yeast 2 hybrid data

**Table S2**. Thermodynamic parameters for all ITC experiments in which binding was observed. All titrations were performed in triplicate.


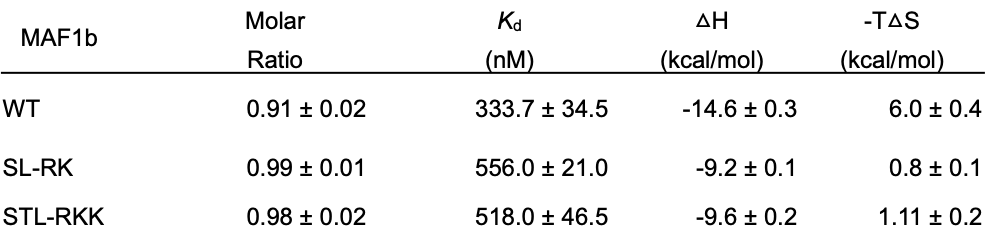


**Table S3**. Hydrogen-deuterium exchange data for MAF1b

**Table S4**. Hydrogen-deuterium exchange data for RalGAPα1(GAP)

**Table S5.** Hydrogen-deuterium exchange source data

**Figures**

**Figure S1.** Schematic of TgMAF1a_WT and mutants (b-loop and RKK-STL) and TgMAF1b_WT and mutants (a-loop and STL-RKK). ITC traces of TgMAF1b mutants are represented in Figure 3.

**Figure S2**: ITC binding isotherm following the titration of TgMAF1a_RKK-STL (left) and TgMAF1a_b-loop (right) into a solution of RalGAPɑ1(GAP).

**
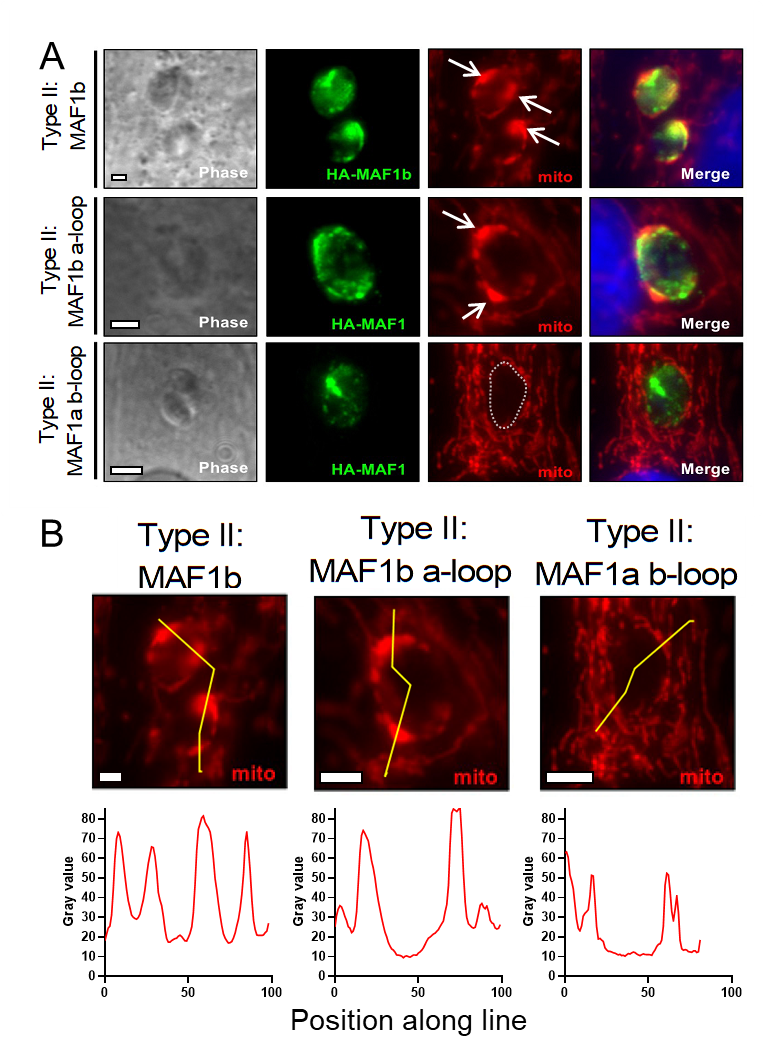
**

**Figure S3: RalGAP⍺1-binding of MAF1b loop is dispensable for HMA.** (A) Impact of mutations on MAF1b-driven HMA. A) HFFs were infected with *Tg*Me49 (Me49∆HPT:Luc) clones of Type II:MAF1b_a-loop and Type II:MAF1a_b-loop. TypeII:MAF1b was used as a positive control for HMA. All infected cells were fixed 24 hpi and visualized using fluorescence microscopy. Immunofluorescence staining was performed with antibodies against the HA epitope tag and an anti-mitochondria antibody (“Mito”: ab110258, Abcam). Arrowheads indicate clear HMA for Type II:MAF1b and Type II:MAF1b_a-loop, while no HMA is evident in Type II:MAF1a_b-loop (PV indicated with dotted line). Scale bars: 5 μm. (B) Images reused from Supplementary figure 3A immediately above. Pixel intensity along the lines indicated were determined using “get profile” in ImageJ Software, showing the increased staining intensity of mitochondria around vacuoles containing parasites expressing WT MAF1b or MAF1b_a-loop, compared to that within vacuoles containing parasites expressing WT MAF1a. Scale bars: 5 μm.


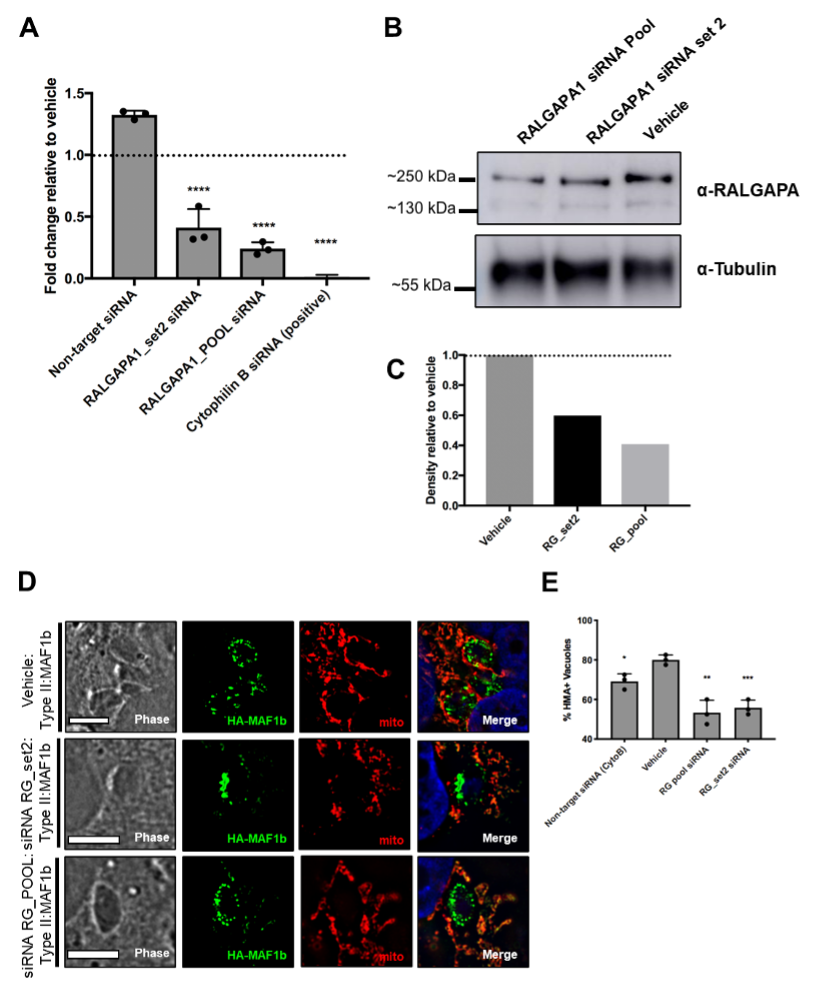


Figure S4 Knockdown of RalGAPα1 protein decreases *T. gondii* association with host mitochondria similarly to siRNA off-target control. (A) U2OS were treated with 40nM of RalGAPα1 (set 2) single target siRNA or RalGAPα1 siRNA smart pool (RG pool) in cDMEM. RNA was collected at 48 hours and qPCR analysis was performed (****p<0.0001, one-way ANOVA multiple comparisons). (B) U2OS cells treated with 40nM of RalGAPα1 siRNA set 2 or RalGAPα1 siRNA pool. At 48 hours, cells were lysed in Pierce IP lysis buffer and boiled in 1x SDS. Samples were resolved on 10% SDS-PAGE gel and transferred to nitrocellulose. Both RalGAPα1 (Sigma-Aldrich Atlas Antibodies #HPA000851) and αTubulin (Cell Signaling Technology #2144) antibodies were used for western analysis. (C) Quantification of pixel intensity density ratio of each treatment RalGAPα1/tubulin bands to vehicle control. (D) U2OS cells were treated as in (A) Following 48 hours of siRNA treatment, cells were infected for 24 hours with Type II: MAF1b parasites (MOI=2). Cells were fixed, permeabilized, and visualized using fluorescence microscopy. Immunofluorescence staining was performed with antibodies against the HA epitope tag and mitochondria. Scale bars: 5 μm. (E) HMA+ and HMA- vacuoles were counted (n=3, 50 per treatment group) using fluorescence microscopy. *p=0.0147, **p=0.0024, ***p=0.0008 unpaired t-test comparing treatment to vehicle.


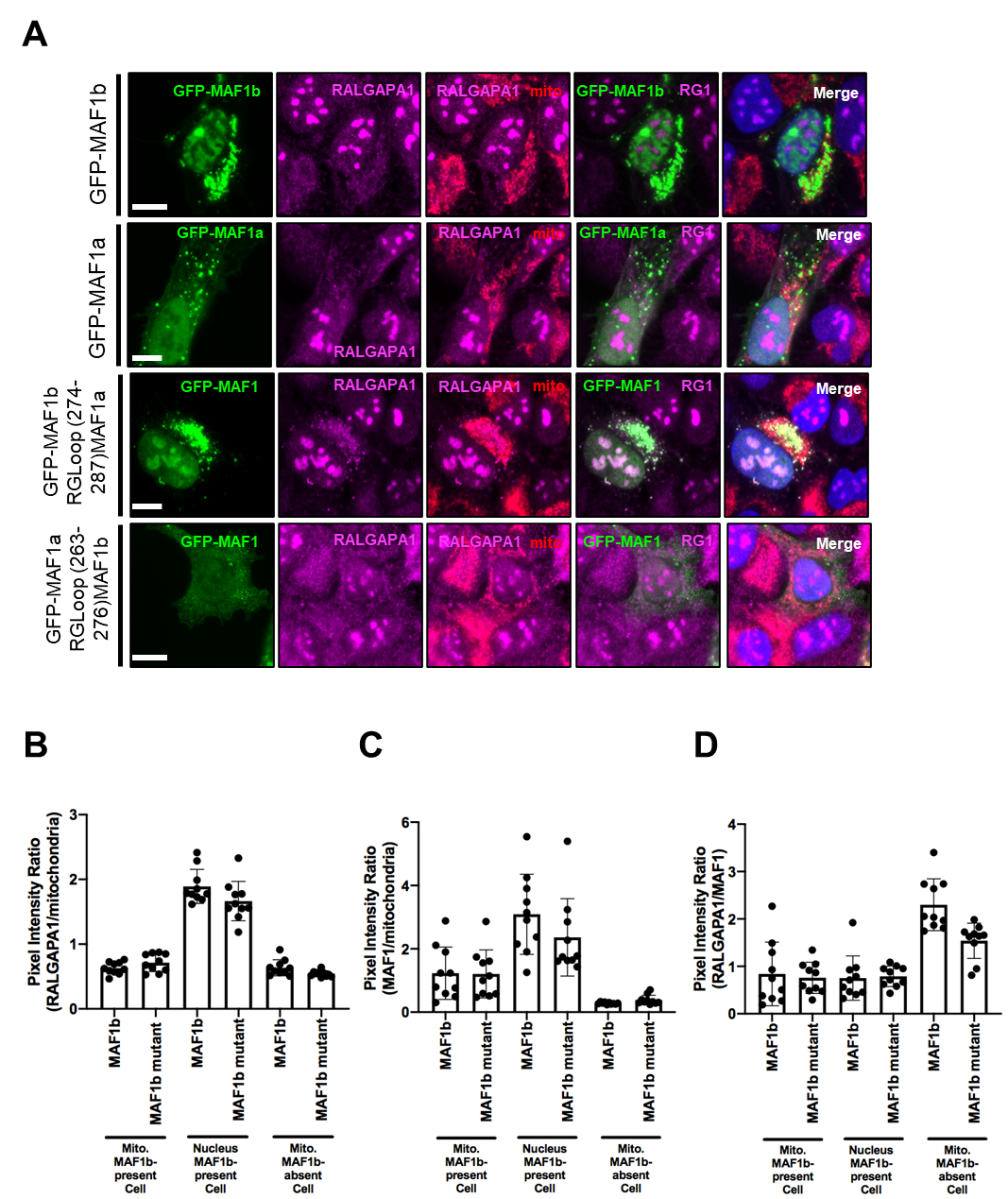


Figure S5 RalGAPα1 localization does not depend on MAF1b localization. (A) Mammalian GFP-MAF1 plasmids were generated using pcDNA3.1/NT-GFP TOPO plasmid cloning kit. U2OS cells were transfected with each construct by lipofection (Lipofectamine 3000, ThermoFisher). Cells were fixed and permeabilized at 24 hours post transfection and stained for RalGAPα1 (Sigma-Aldrich Atlas Antibodies #HPA000851) and mitochondria (Abcam #92824). Scale bars: 10 μm. (B) A selected 15x15 pixel area was chosen for each measurement. Pixel intensity was measured in both the RalGAPα1 and mitochondria channel over the mitochondria of a MAF1-expressing cell, the nucleus of a MAF1-expressing cell, and the mitochondria of a neighboring non-MAF1-expressing cell. The ratio of RalGAPα1/mitochondria pixel intensity was measured for each of these three locations. This same procedure was performed for the MAF1b mutants . (C) Similar to (B), the pixel intensity ratio of MAF1/mitochondria was measured. (D) Similar to (B), the pixel intensity ratio of RalGAPα1/MAF1 was measured.

References

1. Blank, M. L., Xia, J., Morcos, M. M., Sun, M., Cantrell, P. S., Liu, Y., Zeng, X., Powell, C. J., Yates, N., Boulanger, M. J., and Boyle, J. P. (2021) *Toxoplasma gondii* association with host mitochondria requires key mitochondrial protein import machinery. *Proc Natl Acad Sci U S A*. 10.1073/pnas.2013336118
